# *In vitro* evolution predicts emerging SARS-CoV-2 mutations with high affinity for ACE2 and cross-species binding

**DOI:** 10.1371/journal.ppat.1010733

**Published:** 2022-07-18

**Authors:** Neil Bate, Christos G. Savva, Peter C. E. Moody, Edward A. Brown, Sian E. Evans, Jonathan K. Ball, John W. R. Schwabe, Julian E. Sale, Nicholas P. J. Brindle

**Affiliations:** 1 Department of Molecular & Cell Biology, University of Leicester, Leicester, Leicester United Kingdom; 2 Department of Cardiovascular Sciences, University of Leicester, Leicester, Leicester United Kingdom; 3 Leicester Institute of Structural and Chemical Biology, Department of Molecular and Cellular Biology, University of Leicester, Leicester, United Kingdom; 4 School of Life Sciences, The University of Nottingham, Nottingham United Kingdom; 5 Division of Protein & Nucleic Acid Chemistry, MRC Laboratory of Molecular Biology, Cambridge, United Kingdom; Fred Hutchinson Cancer Research Center, UNITED STATES

## Abstract

Emerging SARS-CoV-2 variants are creating major challenges in the ongoing COVID-19 pandemic. Being able to predict mutations that could arise in SARS-CoV-2 leading to increased transmissibility or immune evasion would be extremely valuable in development of broad-acting therapeutics and vaccines, and prioritising viral monitoring and containment. Here we use *in vitro* evolution to seek mutations in SARS-CoV-2 receptor binding domain (RBD) that would substantially increase binding to ACE2. We find a double mutation, S477N and Q498H, that increases affinity of RBD for ACE2 by 6.5-fold. This affinity gain is largely driven by the Q498H mutation. We determine the structure of the mutant-RBD:ACE2 complex by cryo-electron microscopy to reveal the mechanism for increased affinity. Addition of Q498H to SARS-CoV-2 RBD variants is found to boost binding affinity of the variants for human ACE2 and confer a new ability to bind rat ACE2 with high affinity. Surprisingly however, in the presence of the common N501Y mutation, Q498H inhibits binding, due to a clash between H498 and Y501 side chains. To achieve an intermolecular bonding network, affinity gain and cross-species binding similar to Q498H alone, RBD variants with the N501Y mutation must acquire instead the related Q498R mutation. Thus, SARS-CoV-2 RBD can access large affinity gains and cross-species binding via two alternative mutational routes involving Q498, with route selection determined by whether a variant already has the N501Y mutation. These mutations are now appearing in emerging SARS-CoV-2 variants where they have the potential to influence human-to-human and cross-species transmission.

## Author summary

The ability to predict mutations in SARS-CoV-2 that cause increased infectivity or immune evasion would be valuable in development of vaccines and therapeutics, and prioritizing viral variants for monitoring and containment. Using rapid *in vitro* evolution, we identify mutations in SARS-CoV-2 receptor binding domain (RBD) that markedly increase binding of RBD to ACE2. One mutation in particular, Q498H, causes a substantial increase in binding affinity, and we uncover the mechanism for this by structural determination. We show addition of Q498H increases binding of some SARS-CoV-2 variant RBDs but find Q498H is incompatible with the common N501Y mutation. RBD variants with N501Y can instead access similar bonding and affinity increases by acquiring the alternative Q498R mutation. The Q498R/N501Y combination mutations, and Q498H, also enable de novo high affinity binding of SARS-CoV-2 RBD to rat ACE2. Our data show SARS-CoV-2 RBD can access large affinity gains and cross-species binding via two alternative mutational routes involving Q498, with route selection determined by whether a variant already has the N501Y mutation. These mutations are now appearing in new SARS-CoV-2 variants, where they have the potential to contribute to increased variant transmissibility and potentially facilitate cross-species transmission.

## Introduction

SARS-CoV-2 emerged in late 2019 and has led to more than 420 million cases of COVID-19 infection worldwide as of February 2022 [1]. The disease is caused by the betacoronavirus severe acute respiratory syndrome coronavirus-2 (SARS-CoV-2) and arose by zoonotic transmission of the virus from an animal reservoir, most likely horseshoe bat (*Rhinolophus*) via an intermediate host [2]. Since entering the human population, the virus has continued to evolve and adapt to maximise its fitness. This is leading to the emergence of viral variants with increased transmissibility as well as the ability to evade therapeutics and antibodies induced by infection or vaccination [3]. Many of the mutations found in these variants are localised to the viral spike protein, which binds to the host cell receptor angiotensin converting enzyme-2 (ACE2) enabling viral infection [4,5].

In addition to modifying viral transmissibility and immune escape, mutations in the spike protein can also enable the virus to bind ACE2 in species not previously susceptible to infection. Extending the range of species that a virus can infect allows the emergence of new host species and can have important health implications by creating new viral reservoirs with the potential to re-infect the human population. For example, SARS-CoV-2 has been transmitted to mink and spilled back from mink to humans [6]. This potential for cross-species transmission is especially pertinent when viruses infect species that live in close proximity to humans. Importantly, viral evolution in these new reservoirs, and recombination with other coronaviruses, has the potential to create further novel variants, that could potentially cross back into humans.

As the number of SARS-CoV-2 infected individuals rise, in human and other hosts, the opportunities for the appearance of viral mutations resulting in enhanced infectivity or pathogenesis increase. With rising immunity to the virus, there is growing selective pressure on the virus for immune evasion. Consequently, viral variants are emerging with spike protein mutations that enable antibody escape, such as the K417N mutation [3]. Some immune escape mutations lead to a decrease in binding affinity of spike protein for ACE2, and for such mutations, successful viral lineages are those that have combined the escape mutation with additional mutations that restore binding affinity. An example of this is the SARS-CoV-2 B.1.351 (Beta) variant that contains K417N and N501Y mutations. K417N suppresses binding of a number of antibodies [3], but also decreases binding affinity to ACE2 by around five-fold [7,8]. The addition of an N501Y mutation enhances binding affinity and can restore the decrease in affinity caused by K417N [7,8]. With selective pressure for immune escape, mutations that increase binding affinity of the spike protein become more important as they enable the virus to explore a wider range of escape mutations, including those that would otherwise hamper binding to ACE2.

The ability to predict the combinations of mutations that can arise in SARS-CoV-2, and understand how these affect transmissibility and other functions, is critical for development of broadly acting therapeutics and vaccines that will be effective as the virus evolves. This knowledge is also crucial for early identification of variants that should be prioritised for monitoring and containment. Deep mutational scanning of the receptor binding domain (RBD) of SARS-CoV-2 spike protein has provided valuable insights into potential effects of individual mutations affecting stability and affinity for ACE2 [9]. *In vitro* evolution is another powerful methodology that can be used to identify mutations that affect protein activities, and it is particularly effective for revealing combinations of mutations that work together to modify functions.

We have been using a rapid and facile cell surface display evolution approach to seek new mutations and variants of spike protein RBD with functional effects of concern. With this approach we identify an RBD mutant containing S477N and Q498H mutations that exhibits an almost 7-fold increase in binding affinity for human ACE2. We solve the mutant RBD:ACE2 structure to reveal the binding mechanism. The affinity gain of the double mutant is mainly due to the Q498H mutation, and we show this mutation also boosts binding of other RBD variants. Surprisingly however, Q498H inhibits RBD binding if a SARS-CoV-2 variant already has the N501Y mutation, and we find this is due to a clash between the aromatic side chains. In contrast, the related Q498R mutation is compatible with N501Y, and it exhibits positive epistasis with Y501 to enhance binding to ACE2. Importantly, we find that Q498R plus N501Y, as well as Q498H, can also enable variants to bind rat ACE2 with high affinity, opening potential SARS-CoV-2 transmission routes for such variants between humans and rodents. Our data show Q498 to be a pivotal position by which SARS-CoV-2 can access large affinity gains and cross-species binding via two alternative mutational routes, with route selection determined by whether the variant already has the N501Y mutation. These mutations have now been found in new SARS-CoV-2 variants and they may contribute to increased variant transmissibility in the human population and potentially facilitate cross-species transmission.

## Results

**Fig 1:**
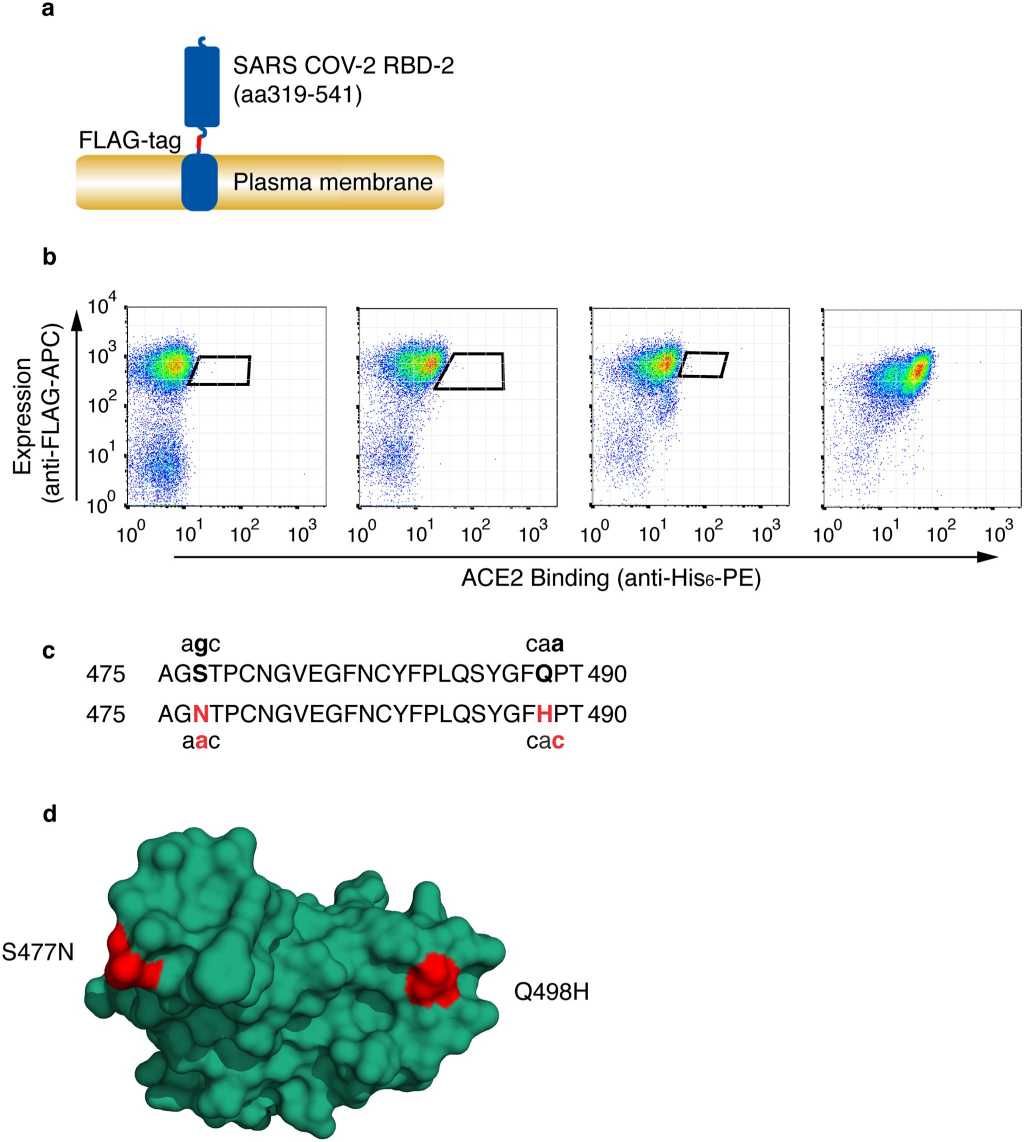
Selection of SARS-CoV-2 receptor binding domain with high affinity for HuACE2. **(a)** Schematic representation of cell surface expressed SARS-CoV-2 RBD showing RBD, FLAG-epitope tag and PDGF-receptor transmembrane anchor. **(b)** SARS-CoV-2 RBD was selected for enhanced ACE2 binding by somatic hypermutation and cell surface display in DT40 cells. FACS plots are shown of DT40 cells following binding of Histidine_6_-tagged-HuACE2. Bound ACE2 was detected with anti-Histidine_6_-phycoerythrin (anti-His_6_-PE) and expression level of RBD was assessed with ant-FLAG-allophycocyanin (anti-FLAG-APC). Sort windows are indicated for each round of selection and diversification, selecting at each round for highest Ang2 binding. Selections were performed at 0.1nM ACE2. The final panel shows a binding plot of the selected cells. **(c)** Amino acid mutations S477N and Q498H in the selected RBD are shown, along with the corresponding single nucleotide mutations. Mutated residues and nucleotides are in red. **(d)** Position of residue mutations in the selected RBD. Mutated residues are shown in red on the ACE2 binding interface (PDB accession number 6M0J [13]). The RBD is orientated to show the face that interacts directly with ACE2.

The DT40 cell surface display system [10,11] was used to identify potential spike protein variants with increased affinity for ACE2. To do this DT40 cells expressing SARS-CoV-2 RBD (residues 19–541) on their surface (Fig 1a) were incubated with the indicated concentrations of monomeric ACE2 (residues 19–615, with a HA epitope tag and C-terminal Histidine_6_) and bound ACE2 detected with anti-Histidine_6_, along with RBD expression level detected using anti-FLAG. Pools of cells with the highest ACE2 binding were selected by taking diagonal sort windows to normalize for RBD expression (Fig 1b). The number of rounds of selection was limited to three to favour selection of variants with a minimal number of accessible mutations, and sort windows were set to capture variants with maximum affinity gain. DNA encoding RBD was amplified by PCR from genomic DNA of the selected cells and sequenced. The dominant variant recovered had the double mutations S477N and Q498H and was designated S477N/Q498H-RBD (Fig 1c). These mutations are located at each end of the binding interface with ACE2 (Fig 1d).

S477N and Q498H mutations have already been reported in isolates from patients. As of February 2022, S477N containing SARS-CoV-2 variants have been found in multiple countries and this mutation has emerged at least seven times during the pandemic [12]. The Q498H mutation is currently much rarer, with 47 genotyped cases being reported in the GISAID database as of February 2022, and around 1.4 million cases with the related Q498R mutation, most of these being in the Omicron variant which also has the S477N mutation [12]. Infections have occurred worldwide. The co-occurrence of S477N and Q498H has yet to be reported.

### SARS-CoV-2 RBDs with Q498H mutations have high affinity for ACE2

**Fig 2:**
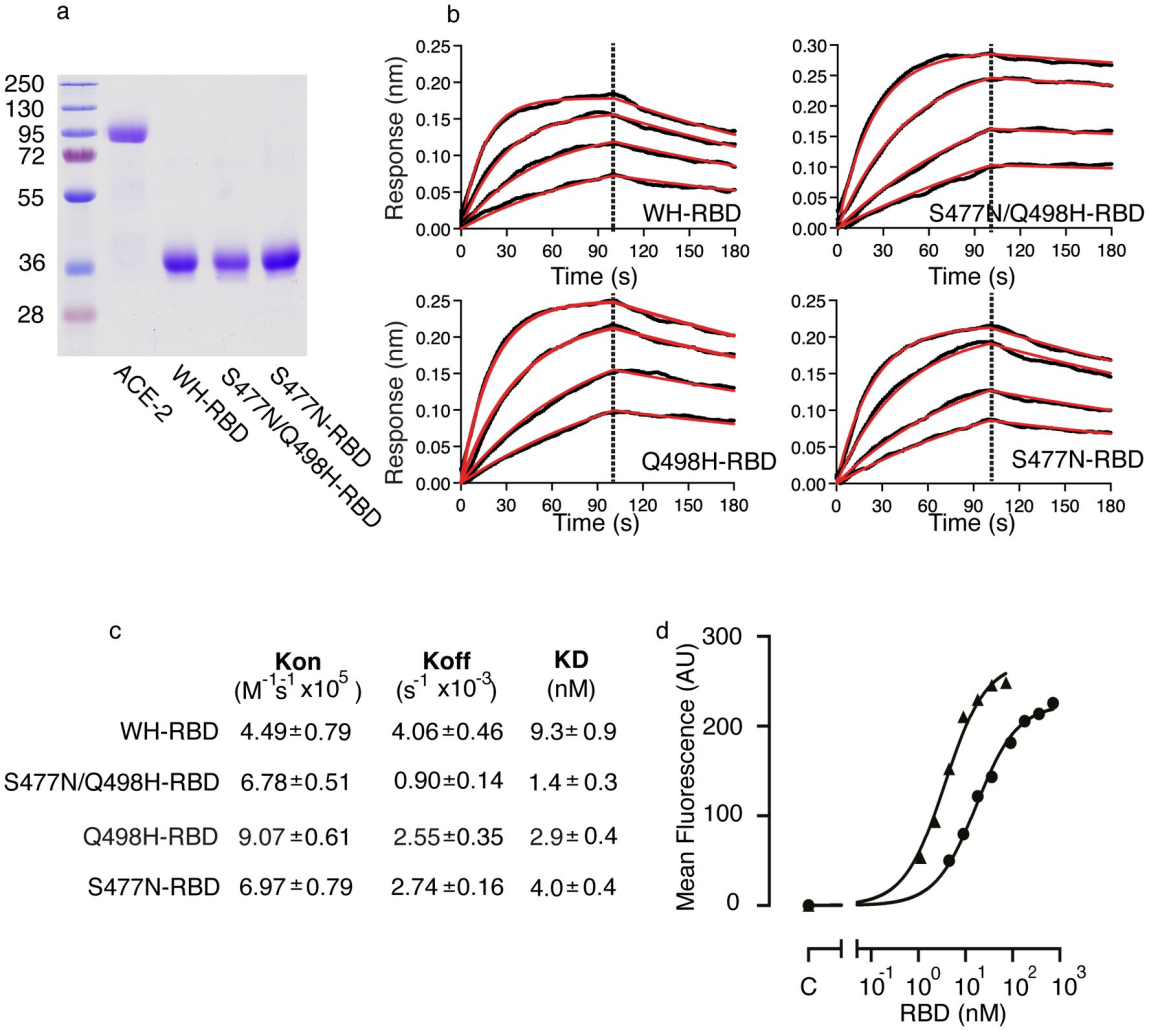
SARS-CoV-2 RBDs with Q498H mutations have high affinity for ACE2. **(a)** Soluble forms of HuACE2, WH-RBD and mutant RBD were expressed in HEK 293 cells and purified on nickel affinity columns. Purified proteins were resolved by SDS/PAGE and detected by Coomassie staining. Molecular masses are shown in kDa. **(b)** Biolayer interferometry plots of kinetics of binding RBD to HuACE2. WH-RBD, or the indicated RBD, was in solution and Hu-ACE2 immobilized on sensors. Binding was measured with RBD concentrations of 12, 30, 60, 120nM for WH-RBD and 6, 12, 30, 60nM for the mutants. Fitted curves are in red. Data are shown for single experiments representative of at least three independent experiments for each RBD. **(c)** Kinetic binding constants for RBD binding to HuACE2 measured using biolayer interferometry. Data are shown as means and SEM for at least three independent experiments. **(d)** Cellular binding of RBD and S477N/Q498H-RBD. Vero-E6 cells were incubated without RBD (C) or with a range of concentrations of WH-RBD (circles) or S477N/Q498H-RBD (triangles) as indicated, at 37°C for 15 mins before washing, antibody staining of bound RBD with fluorescently conjugated antibody, and flow cytometry. Data are shown as mean fluorescence intensity vs RBD concentration (nM) for a single experiment representative of three.

The binding ability of the soluble epitope-tagged RBD of Wuhan-Hu-1 SARS-CoV-2 (WH-RBD) and mutant RBD (Fig 2a) was measured using biolayer interferometry (BLI) with human ACE2 (Hu-ACE2) immobilized on the biosensor (Fig 2b). In our experiments WH-RBD binds Hu-ACE2 with a K_D_ of 9.3nM, whereas S477N/Q498H-RBD bound Hu-ACE2 with a K_D_ of 1.4nM, representing around 6.5-fold higher affinity of binding than WH-RBD (Fig 2b and 2c). To assess the contribution of each mutation to the overall increase in affinity, we assayed the Q498H and S477N mutations individually. RBD with Q498H bound ACE2 with a K_D_ of 2.9nM. The S477N mutant was found to bind ACE2 with a K_D_ of 4.0nM (Fig 2b and 2c). In S477N/Q498H-RBD, N477 and H498 are positioned at each end of the RBD binding interface and work together to stabilize the RBD:ACE2 complex, once formed, as indicated by the more than 4-fold decrease in K_off_ observed for the combined mutant (Fig 2b and 2c).

To test whether the increase in binding of S477N/Q498H-RBD for ACE2 seen in BLI assays is reflected in an increase in binding affinity in the cellular context we used flow cytometry (Fig 2d). Vero-E6 cells, which express ACE2, were incubated with varying concentrations of WH-RBD or S477N/Q498H-RBD, cells were washed and bound RBD was detected with a fluorescent antibody recognizing the epitope tag on the RBD proteins. The mean fluorescence intensity of the cells was measured by flow cytometry and plotted against RBD concentration. Both WH-RBD and S477N/Q498H-RBD bound in a concentration dependent manner (Fig 2d). The concentration of WH-RBD required for half-maximal binding was 17.4 +/- 0.3nM compared with 4.3 +/- 0.4nM (mean and SEM for three independent experiments) for S477N/Q498H-RBD. Consistent with the data on *in vitro* binding to purified ACE2, therefore, S477N/Q498H-RBD bound with higher apparent affinity than WH-RBD.

### Structure of S477N/Q498H-RBD in complex with HuACE2

**Fig 3:**
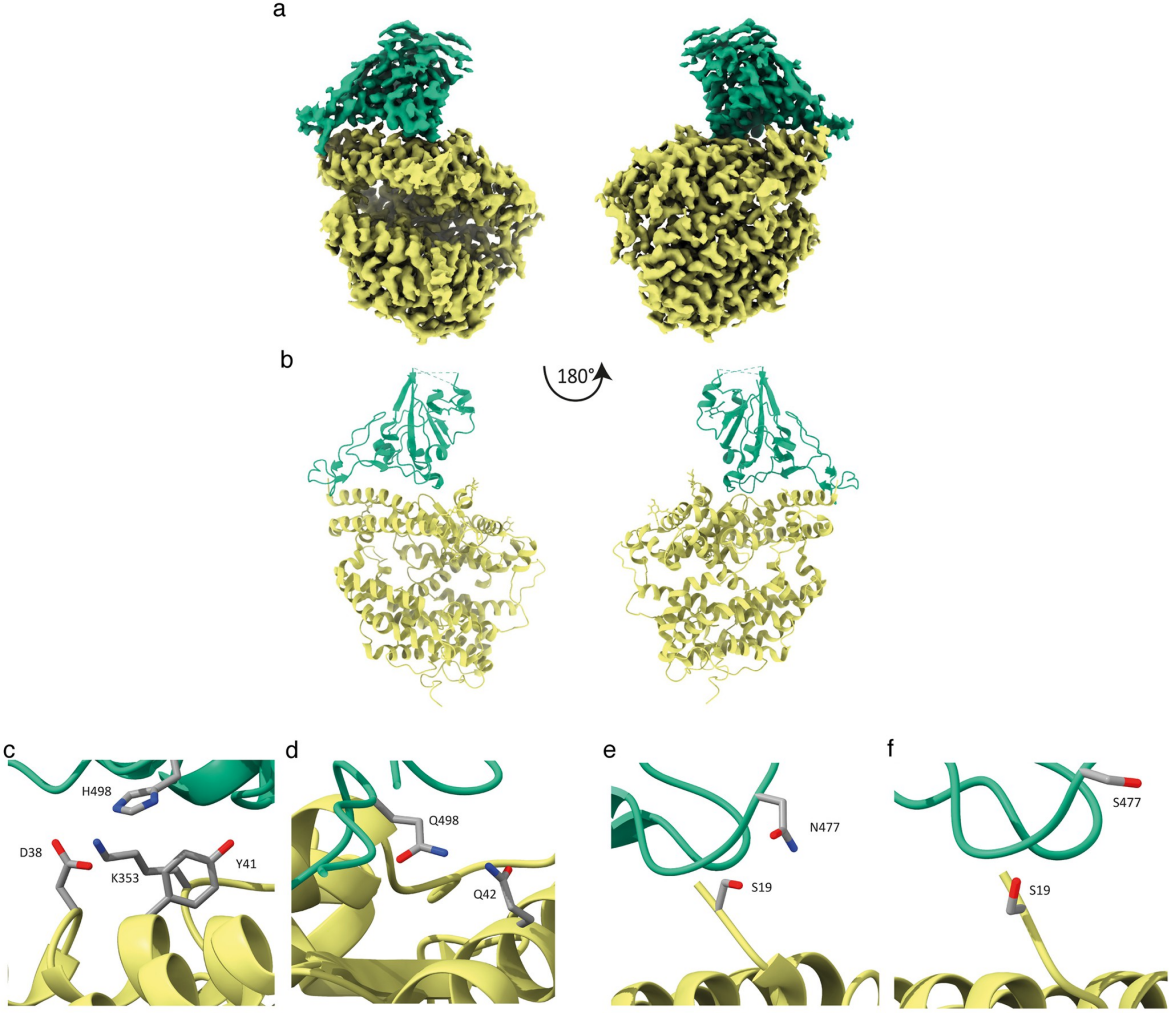
Structure of S477N/Q498H-RBD in complex with HuACE2. Cryo-EM structure and model of the ACE2-RBD complex. Sharpened Cryo-EM map **(a)** of the HuACE2-S477N/Q498H-RBD complex with ACE2 coloured yellow and RBD in green. The refined coordinates shown in cartoon representation **(b)** and coloured as above. In panel **(c)** H498 can be seen in proximity to ACE2 Y41 forming a non-planar π-interaction while ACE2 residues K353 and D38 are within hydrogen bonding distance to H498 and could contribute to the tighter interaction formed by this RBD mutant, whereas Panel **(d)** shows Q498 in WH-RBD (PDB accession number 6M0J [13]) in proximity to ACE-2 Q42. Panel **(e)** indicates that the S477N mutation places this longer side-chain closer to S19 in ACE2 and within hydrogen bonding distance thus enhancing the binding between HuACE2 and S477N/Q498H-RBD. Panel **(f)** shows positioning of S477 in WH-RBD and ACE2 S19 (PDB accession number 6M0J [13]).

The increase in affinity of S477N/Q498H mutated RBD, and particularly the substantial increase in binding due to the Q498H mutation, prompted us to determine the structure of the S477N/Q498H-RBD:ACE2 complex to gain insight into the binding mechanism. The complex was imaged by Cryo-EM to produce a good quality 3.2Å map for which we could build an atomic model for the complex and observe the new interactions across the binding interface (Fig 3a and 3b).

Examination of the structure reveals mutation of RBD Q498 for histidine results in a perpendicular Y-shaped π-interaction between ACE2 Y41 and RBD H498 (Fig 3c). In addition, RBD H498 now lies within hydrogen bonding distance of a flexible ACE2 lysine residue (K353) which would strengthen the interaction in this area. A neighbouring aspartate side chain (ACE2 D38) could also mediate a hydrogen bond via a water molecule to RBD H498 and this is supported by a continuous density between ACE2 D38 and RBD H498 at lower contour levels. Although we are unable to build water molecules into the structure at this resolution, we do find several densities in our map coincide exactly with the water molecules built for the x-ray model used as starting coordinates for this study (PDB 6M0J; [13]). On the other side of the interface between ACE2 and RBD the gain of a carbon atom from the RBD S477N mutation places this side chain in hydrogen bonding distance of ACE2 S19 (Fig 3e), whereas S477 in the un-mutated RBD is unable to reach S19 (Fig 3f).

In addition to the newly formed interactions, we examined how the new binding interface may be further favourable over the Wuhan-Hu-1 interface. Examination of the difference electron density map of PDB 6M0J [13] reveals that although RBD Q498 can hydrogen bond with ACE2 Q42 (Fig 3d), the positive difference electron density adjacent to the side chain is consistent with disorder (i.e. it adopts at least one alternate conformation) (S1 Fig). Alternate conformations would break the hydrogen bond with ACE2 Q42 suggesting that the contribution of RBD Q498 to ACE2 binding is reduced. This disorder in Q498 can be seen in all but one of the crystal structures in the PDB.

### Q498H and Q498R mutations modify binding of B.1.617.1/3 and B.1.351 variant RBD to HuACE2

**Fig 4:**
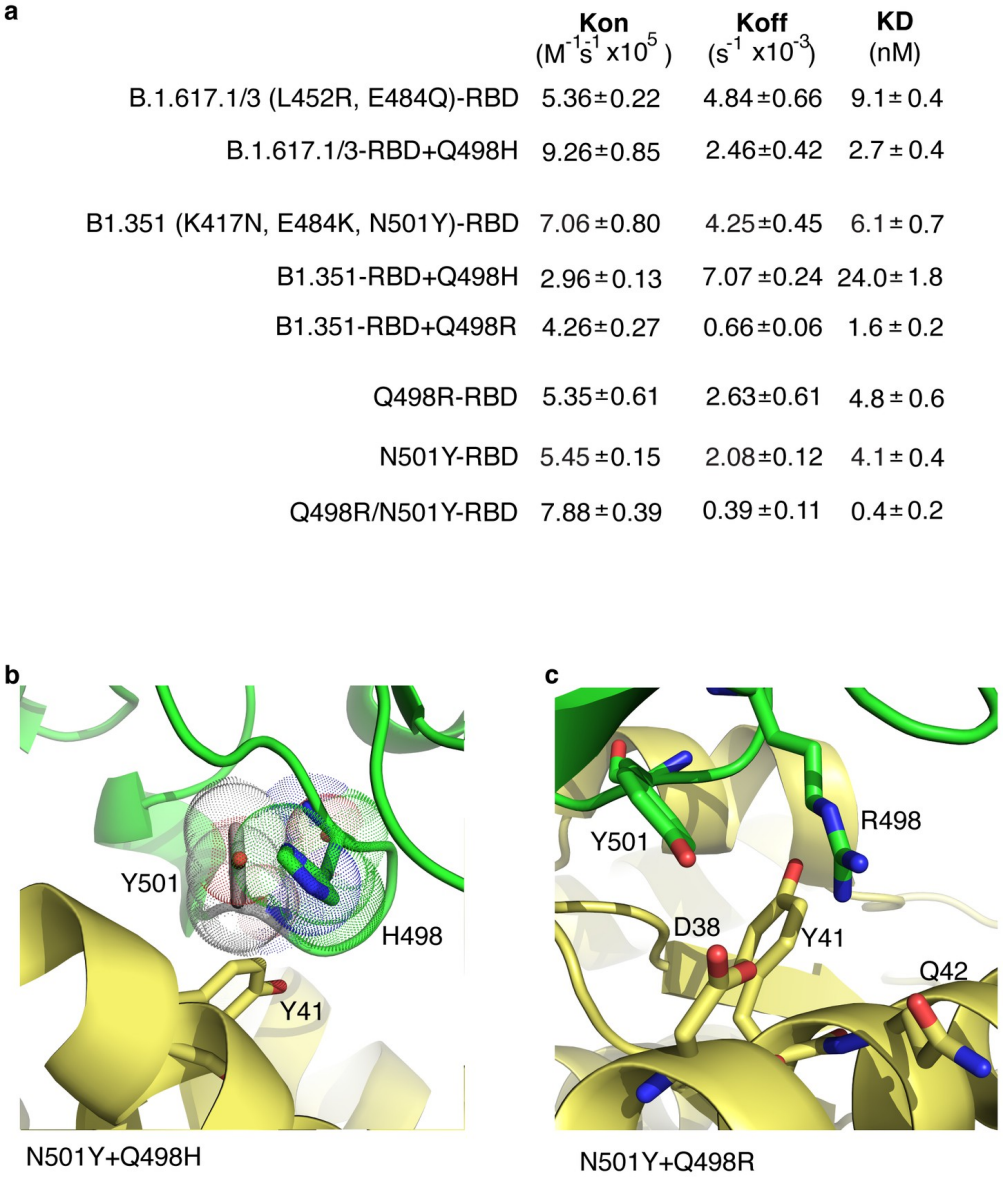
Q498H and Q498R mutations modify binding of B.1.351 and B.1.617.1/3 variant RBD to HuACE2. **(a)** Biolayer interferometry was performed with the indicated RBD in solution and HuACE2 immobilise on the sensor. Binding was measured with RBD concentrations of 12, 30, 60, 120nM, and an additional 6nM concentration in some cases. Curves were fitted and used to calculate K_on_, K_off_ and K_D_. Data are shown as means and SEM for at least three independent experiments. **(b)** H498 and Y501 in SARS-CoV-2 RBD compete for interaction with Y41 in HuACE2 and the clash between residues is indicated by the overlapping Van der Waals surfaces. The proximity of the H498 side chain is shown with respect to the side chain of a Y501 inserted into our RBD:ACE2 complex structure. **(c)** R498 and Y501 side chain positions shown along with D38, Y41 and Q42 (PDB accession number 7BH9 [14]).

The finding that mutating histidine at position 498 in SARS-CoV-2 RBD causes a dramatic increase in binding affinity for ACE2 prompted us to test whether this mutation could enhance binding of other SARS-CoV-2 RBD variants. We therefore added the Q498H mutation to two prominent RBD variants, one containing the mutations L452R and E484Q, which are common to variants B.1.617.1 (Kappa) and B.1.617.3 (Delta), and one containing RBD mutations K417N, E484K and N501Y, found in variant B.1.351 (Beta). Addition of Q498H to B.1.617.1/3 increased affinity by around three-fold for ACE2, decreasing K_D_ from 9.1nM to 2.7nM (Fig 4a).

Surprisingly, addition of Q498H to the B.1.351 variant RBD markedly reduced the affinity of B.1.351 (Fig 4a). As shown in Fig 4b, H498 and Y501 are in close proximity and the local arrangement required to accommodate the phenolic group of Y501 will be constrained by the imidazole of H498, reducing the advantageous π-π stacking between Y501 and Y41 in B.1.351 as well as perturbing the H498-Y41 interactions. Thus, the negative epistasis between Y501 and H498 results from this conflict of their side chain ring structures. As introduction of a histidine at position 498 clashes with Y501, we tested the effect of an arginine mutation at 498 (Fig 4a), as this is also a basic residue and the Q498R mutation is found in some SARS-CoV-2 variants [12]. Addition of Q498R to the N501Y-containing variant increased affinity 3.8-fold, yielding an K_D_ of 1.6nM. This increase in affinity is due to a large decrease in off-rate, indicating the Q498R mutation in the N501Y variant acts to stabilize binding to ACE2. The increase in affinity found on addition of Q498R to the N501Y-containing variant indicates that arginine can be accommodated at position 498 without clashing with Y501, allowing both Y501 and R498 to contribute to binding. Indeed, in the absence of any other mutations RBD with the double mutation Q498R plus N501Y has very much higher affinity for ACE2 than RBD with either N501Y alone or Q498R alone (Fig 4a). To gain insight into the molecular mechanism for the affinity gain afforded by the N501Y plus Q498R mutations we examined a previously published RBD:ACE2 structure [14] in which RBD has Q498R and N501Y mutations, in addition to several other mutations. This structure suggests RBD Y501 makes a π-interaction with ACE Y41 and RBD R498 forms a hydrogen bond with ACE Q42 and a potential salt bridge with ACE D38, and could also form a cation- π interaction with Y41 (Fig 4c). Therefore, the mechanism by which the combined mutations of Q498R plus N501Y increases affinity has similarities to that by which the single Q498H mutation increases affinity.

Overall, these data show that the affinity of B.1.617.1/3 variant RBD for ACE2 is enhanced by the addition of the Q498H mutation. In contrast, in a variant which already has an N501Y mutation, the Q498H mutation clashes structurally with Y501 and fails to increase affinity. However, arginine can be accommodated at 498 in a N501Y variant, and this results in a large affinity gain by a bonding mechanism requiring both R498 plus Y501, that shares similarities with that of H498 alone.

### The Q498H mutation enables RBD binding to rat ACE2

**Fig 5:**
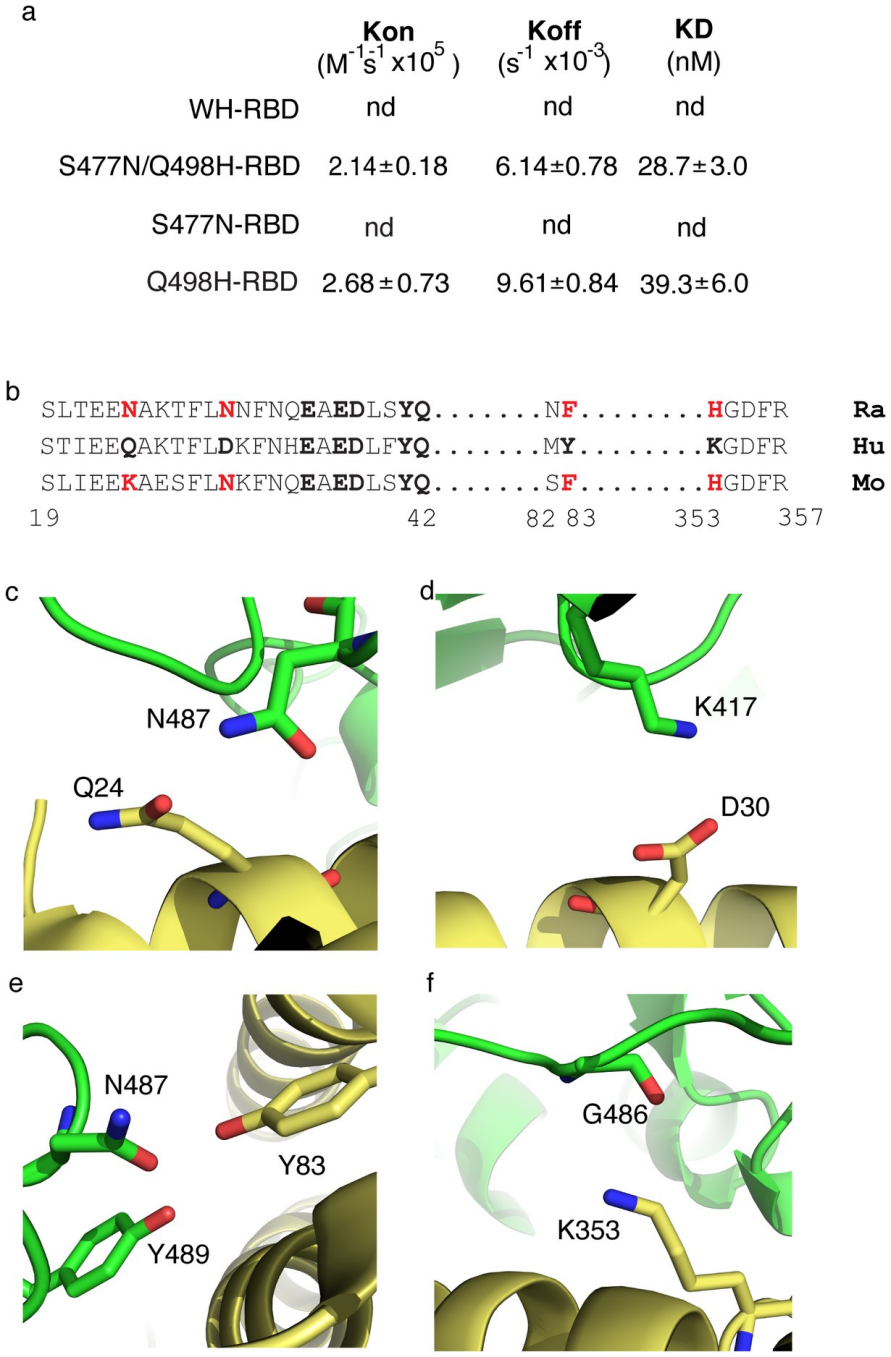
The Q498H mutation enables binding of SARS-CoV-2 RBD to rat ACE2. **(a)** Kinetic binding constants for RBD binding to RaACE2 measured using biolayer interferometry. Binding was measured with RBD concentrations of 48, 90, 150, 300 and 600nM. Where no binding was detected an additional 1200nM RBD concentration was also tested for binding. RBD with the Q498H mutation binds rat receptor. Data are shown as means and SEM for at least three independent experiments. **(b)** Rat (RaACE2) and mouse ACE2 (MoACE2) differ from human ACE2 (HuACE2) in key residues contributing to SARS-CoV-2 RBD binding. Residues in human ACE2 at the RBD binding interface are aligned with the corresponding residues in rat and mouse ACE2. Residues that form hydrogen bonds or salt bridges with RBD are in bold and those that differ in rodent are in red. (**c**) Bonding interactions between Q24 and N487 (**d**) D30 and K417 (**e**) Y83 and N487 plus Y489, and (**f**) K353 and G496, in HuACE2 (yellow) and RBD (green).

RBD of SARS-CoV-2 Wuhan-Hu-1 is unable to bind rodent ACE2. However, in a recent study to develop a mouse model of SARS-CoV-2 infection, SARS-CoV-2 was passaged multiple times through mice to derive a variant capable of murine infection [15]. The resultant variant had two mutations in RBD, Q493K and Q498H, and was capable of binding to mouse ACE2 and causing SARS-CoV-2 infection [15]. Interestingly, either of these mutations alone appears sufficient to allow RBD to bind mouse ACE2. This finding suggests that SARS-CoV-2 variants that include a Q498H mutation would be able to bind mouse ACE2 and allow the virus to extend its species range of infectivity. We therefore tested whether the high affinity RBD variant S477N/Q498H-RBD can bind rodent ACE2. Given the similarity between mouse and rat ACE2 (RaACE2) in the region corresponding to the SARS-CoV-2 binding site (Fig 5b), and the importance of rats as a potential viral reservoir, we focussed on testing binding to rat ACE2.

Like the situation with mouse ACE2, we could not detect any binding between WH-RBD and RaACE2 (Fig 5a). However, we found that S477N/Q498H-RBD was clearly capable of binding RaACE2, with a K_D_ of 28.7nM, which is comparable to the affinity of Wuhan-Hu-1 RBD for human ACE2 (Fig 2c). RBD with the S477N mutation alone was unable to bind the rodent receptor, whereas RBD with just the Q498H mutation bound with an affinity of 39.3nM (Fig 5a).

### Mechanisms of binding to rodent ACE2

To understand why Wuhan-Hu-1 can bind human ACE2 but not the rodent receptor, we identified which HuACE2 residues involved in RBD binding differ in rat ACE2. Binding of RBD to HuACE2 involves three key regions of ACE2, the alpha helix between residues 19 and 42, residues 82 and 83 and residues 353 to 357 [13,16,17]. Alignment of the ACE2 sequences encompassing these regions for human and rat reveals differences between the species (Fig 5b). Four residue positions in HuACE2 identified as being involved in salt bridge or H bonding with SARS-CoV-2 RBD differ in the rat sequence, namely Q24, D30, Y83 and K353 in HuACE2 vs N24, N30, F83 and H353 in RaACE2 (Fig 5b). Of these, Q24 in HuACE2 hydrogen bonds with RBD N487 (Fig 5c), and this hydrogen bond will also likely be formed with N24 in the rat. In contrast, D30 in HuACE2 forms a salt bridge with RBD K417 (Fig 5d) and this would not occur with the corresponding N30 in rat ACE2. Y83 in HuACE2 forms two hydrogen bonds with N487 and Y489 (Fig 5e) and these would also be abolished in rat ACE2 where position 83 is a phenylalanine. K353 in HuACE2 hydrogen bonds with G496 (Fig 5f), however position 353 in RaACE2 is a histidine and the shorter reach and lower mobility of the histidine side chain would limit its to hydrogen bond with G496. Overall, the one fewer salt bridge and at least two fewer H bonds that RaACE2 could form with WH-RBD, compared with HuACE2 binding to WH-RBD, could result in the different abilities of rat and human ACE2 to bind WH-RBD. Like rat, mouse ACE2 also has N30, F83 and H353 (Fig 5b) and therefore, as with RaACE2, would form one fewer salt bridge and at least two fewer hydrogen bonds than can HuACE2 with WH-RBD, which could account for the lack of detectable binding of mouse ACE2 to WH-RBD.

The ability of Q498H RBD to bind RaACE2 indicates RaACE2 can form inter-molecular bonds with H498 that are not available to Q498 in WH-RBD. With HuACE2, H498 forms bonds with D38, Y41 and K353, leading to the increase affinity of the H498 mutant over WH-RBD for the human receptor (see above). D38 and Y41 are also present in rat ACE2 (Fig 5b). Therefore, the H498 could form a π-interaction with RaACE2 Y41 and a hydrogen bond with RaACE2 D38, as in HuACE2, providing additional binding energy for the Q498H binding. In addition, in HuACE2, K353 makes an intramolecular salt bridge with D38 [13]. The shorter H353 in RaACE2 would not be able to do this, leaving D38 available for possible formation of a salt bridge with H498 in the mutant RBD. These new bonding possibilities afforded by H498 in the mutant RBD could at least partially compensate for the bonds lacking from N30 and F83 in RaACE2, bringing binding of the mutant RBD to RaACE2 up to a detectable level. D38, Y41 and H353 are also conserved in mouse ACE2 (Fig 5b), potentially allowing H498 to form similar interactions and enable the RBD to bind murine ACE2 [15].

### Q498H and Q498R + N501Y enable binding of RBD variants to rat ACE2

We were interested to test whether B.1.617.1/3 and B.1.351 variant RBDs can bind RaACE2, and whether this is affected by acquisition of an additional basic mutation at Q498. As shown in Table 1 we found no detectable binding of the B.1.617.1/3 variant RBD to RaACE2. However, addition of the Q498H mutation increases affinity for RaACE2 resulting in detectable binding of this variant, with a K_D_ of 23.3nM. RBD variant B1.351 did not bind detectably to RaACE2 (Table 1). As mutation Q498H was found to clash with N501Y (see above), we tested the effect of addition of Q498R to the B1.351 RBD, and found the additional mutation increases binding to detectable levels, resulting in a K_D_ of 38.6nM for RaACE2. This binding to RaACE2 is afforded by Y501 and R498 acting in combination, as in the absence of any other mutations binding to RaACE2 is below detectable levels for RBD with either Y501 alone or R498 alone, whereas RBD with both mutations together can clearly bind the rat receptor (Table 1).

**Table 1:** Effects of Gln498His and Gln498Arg mutations on binding of B.1.617.1/3 and B.1.351 variant RBD to RaACE2.

RBD Mutations	Kon (M^-1^S^-1^ x 10^5^)	Koff (S^-1^ x 10^−3^)	KD (nM)
B.1.617.1/3 (L452R, E484Q)	nd	nd	nd
B.1.617.1/3 + Q498H	3.05 ± 0.51	6.71 ± 0.21	23.3 ± 3.7
B.1.351 (K417N, E484K, N501Y)	nd	nd	nd
B.1.351 + Q498R	2.76 ± 0.30	11.1 ± 0.45	38.6 ± 2.9
Q498R	nd	nd	nd
N501Y	nd	nd	nd
Q498R + N501Y	3.02 ± 0.59	13.8 ± 1.2	52.4 ± 5.5

Biolayer interferometry was performed with the indicated RBD in solution and RaACE2 immobilised on the sensor, nd indicates signal not detectable. Binding was measured with RBD concentrations of 48, 90, 150, 300 and 600nM. Where no binding was detected an additional 1200nM RBD concentration was also tested for binding. Data are shown as means and SEM for at least three independent experiments.

## Discussion

In this study we have used a rapid and facile directed evolution system to discover mutations that could arise in SARS-CoV-2 RBD leading to large increases in binding affinity for ACE2. We identify a double mutant form of SARS-CoV-2 spike protein RBD (S477N/Q498H) with a marked enhancement in binding affinity for HuACE2. Furthermore, we show that the Q498H mutation, and the related Q498R mutation plus N501Y, can enable *de-novo* binding of RBD to rat ACE2. We provide a molecular mechanism for the increased affinity of the double mutant and also show that Q498H, or the related Q498R mutation, confers large affinity gains on other SARS-CoV-2 RBD variants. The Cryo-EM structure of the S477N/Q498H-RBD:ACE2 complex revealed the newly formed interactions of a histidine at position 498, including π-stacking and hydrogen bonding to neighbouring ACE residues, strengthens the previous transient interactions between Q498 and ACE2. In addition, a new hydrogen bond between N477 and ACE2 at the other end of the RBD binding interface contributes to the almost 7-fold binding increase. Nonetheless, the increase in binding is driven mainly by the Q498H. Indeed, Histidine at position 498 results in a substantial affinity gain. Consistent with this, Starr *et al* [9], using deep mutational scanning, show Q498H to be the most enriched mutation in their highest binding RBD pool.

We hypothesised that the Q498H mutation would increase binding affinity of other SARS-CoV-2 variant RBDs, and indeed gain of Q498H resulted in a clear enhancement of binding in B.1.617.1/3 variant RBD. However, in contrast addition of Q498H to B.1.351 variant RBD decreases affinity, raising the K_D_ for ACE2 from 6.1nM to 24.0nM (Table 1). The reason for this is evident from structural analysis, which reveals close proximity of H498 to the Y501 mutation in B.1.351, causing a clash of the side chain rings for binding to ACE2 residues. However, we find that a variant RBD with tyrosine at 501 can accommodate an arginine at 498, enhancing affinity. Interestingly, Q498R results in a smaller increase of 2-fold in affinity in the absence of a tyrosine at 501 in RBD. This suggests Q498R mutations in SARS-CoV-2 RBD would be more likely in lineages in which N501Y is already fixed. Indeed, in a recent RBD *in vitro* evolution study, the Q498R mutation was only evident after early fixation of N501Y and high selection pressure for affinity [14].

Our data show SARS-CoV-2 RBD, therefore, can gain a substantial affinity boost by two different routes both involving position 498, and mutation preferences between Q498H and Q498R are determined by whether N501Y is already present in the RBD. SARS-CoV-2 RBD lineages with an N501Y mutation would be unlikely to gain Q498H without losing binding fitness, whereas such variants could gain Q498R leading to increased affinity of RBD. Significantly more than 85% of reported SARS-CoV-2 variants containing the Q498R mutation also have the N501Y mutation, and the vast majority of these are the recently described B.1.1529 variant (Omicron), that also contains S477N and a range of other mutations [18]. This variant is expanding rapidly and based on our findings, we suggest that Q498R plus N501Y, as well as S477N, could contribute to the binding ability of Omicron, and other variants containing these mutations. Our findings also mean that SARS-CoV-2 lineages without N501Y in RBD could potentially gain a corresponding substantial affinity increase by acquiring just the single Q498H mutation, raising the possibility of parallel viral lineages with either Q498H or Q498R plus N501Y as affinity contributors.

Interestingly, several coronaviruses (CoVs) isolated from animals and highly related to SARS-CoV-2 have histidine at residues equivalent to 498, including CoVs from pangolins [19, 20]. We have shown here that H498 in SARS-CoV-2 RBD contributes to high affinity binding to HuACE2 by forming new bonding interactions with a number of residues in the human receptor. It is possible, therefore, that this residue could contribute to HuACE2 binding in some of the related CoVs from pangolin and other species. However, others have clearly shown animal CoVs require a sufficiently high structural and sequence similarity in their RBDs to SARS-CoV-2 RBD to bind the human receptor [21–23]. Therefore, although H498 in animal CoVs could contribute to HuACE2 binding, additional key residues would also be needed in the CoV RBD and the domain would need to be appropriately structurally configured for binding to occur.

Ordinarily, Wuhan-Hu-1 SARS-CoV-2 RBD is unable to bind detectably to rodent ACE2 and here we show why this is the case. However, we find that mutations at Q498 opens up RBD binding to rat ACE2, our analysis pinpointing the D38, Y41 ACE2 residue dyad as a key site exploited by the virus for the enabling affinity gain. Addition of variant-specific Q498 mutations also confer rat ACE2 binding on SARS-CoV-2 RBD variants. In our experiments, gaining a Q498H, or Q498R plus N501Y, resulted in binding of RBD variants that were otherwise incapable of detectable binding to rat ACE2. These mutations are already found in emerging SARS-CoV-2 variants, and thus Q498H, or Q498R plus N501Y, have the potential to extend transmission of variants into rodent populations. Acquisition of mutations that enable SARS-CoV-2 to bind rat ACE2 is a cause of concern, as this has the potential to facilitate transmission of the virus to a species that is widespread and lives close to humans. Although, whether interactions between humans and rats would be sufficient to allow cross-species transmission is not yet known. Further evolution in such a reservoir carries the additional potential risk of a spill back to humans of novel variants with further detrimental phenotypes. Worryingly, SARS-CoV-2 variants with Q498H, and Q498R plus N501Y, have already been detected in wastewater [12], a potential transmission route to rat.

It should be noted that the present study focusses on mutations in the RBD. Whilst we report mutations that modify binding of RBD to ACE2, additional work will be required to determine whether these mutations lead to effects on viral binding or infectivity. Specifically, it will be necessary to test whether the mutations cause corresponding changes in binding affinity in the context of full-length spike protein, and intact virus, as well as directly testing their effects on virus infectivity, using for example pseudotyped virus entry assays.

## Materials and methods

### Materials

DNA encoding the N-terminal secretory leader sequence from CD5 (residues 1–24) upstream of either HuACE2 (residues 19–615) or RaACE2 (residues 19–165), followed by a short linker FLAG-epitope tag and C-terminal Histidine_6_ was synthesised by GeneArt (Invitrogen). ACE2-Fc constructs include a GS4-linker and fragment of human IgG1 Fc (residues 104–330) between ACE2 and FAG-epitope. To generate a FLAG-free HuACE2 fusion, the FLAG-epitope tag in the HuACE2 construct was replaced with a HA tag. Constructs encoding soluble RBD proteins comprise of DNA encoding the N-terminal secretory leader sequence from CD5 (residues 1–24) upstream of Wuhan-Hu-1 SARS-CoV-2 RBD (residues 319–541), or RBD mutants indicated in Results, followed by a short linker HA-epitope tag and C-terminal Histidine_6_ and were synthesised by GeneArt (Invitrogen). All other reagents were as described previously [11,24].

### Cell surface display

The cell surface display DT40 system that we previously described [10] was used. cDNA encoding a fusion protein comprising of an N-terminal CD5 secretory leader sequence followed by the Wuhan-Hu-1 SARS-CoV-2 RBD (residues 319–541), linker region and FLAG epitope tag together with a C-terminal transmembrane domain and short intracellular domain fragment of platelet-derived growth factor receptor-β was synthesised (GeneArt Gene Synthesis) and inserted into the pHypermut2 vector [25] This was transfected into DT40 cells by electroporation and stable transfectants derived by growth in puromycin. Clonal DT40 lines in which the RBD construct was integrated into the rearranged Ig locus were identified by PCR, and surface expression of the fusion construct confirmed by anti-FLAG immunostaining as previously described [10]. Cells were cultured in RPMI containing 7% (v/v) fetal bovine serum (FBS) plus 3% (v/v) chicken serum at 37°C and 5% CO_2_.

For ACE2 binding to DT40 cells, approximately 40 million cells were washed and incubated with 0.1nM Histidine_6_-tagged HuACE2 in PBS with 10% (v/v) FBS at room temperature for 30 mins. Cells were washed and incubated with anti-FLAG-allophycocyanin and anti-Histidine_6_-phycoerythrin antibodies on ice for 20 mins, followed by washing. Cells with bound ACE2 were selected by fluorescence activated cell sorting on a FACS Aria Fusion (Becton Dickinson) with the sort windows indicated in Results. Sorted cells were resuspended and grown in DT40 culture medium. For sequencing, genomic DNA from an aliquot of the sorted cell population was recovered using PureGene DNA isolation kit (Qiagen) and RBD amplified by PCR. Purified PCR products were directly sequenced. In addition, purified PCR products were inserted into pcDNA3.1, transformed into *E*. *coli* and sequencing was performed on randomly picked colonies.

### Site-directed mutagenesis and soluble Fc-fusion proteins

Site directed mutagenesis was performed using the QuikChange protocol (Agilent Technologies) and constructs were sequenced to confirm mutations. cDNA encoding Fc-fusion proteins were constructed by ligating the appropriate RBD or ACE2 nucleotide sequence upstream of a GS4 linker, fragment of human Fc immunoglobulin domain and C-terminal Histidine_6_ tag.

### Expression and purification of soluble proteins

HEK 293 cells were transfected with mammalian expression vectors encoding the relevant protein using polyethylenimine and cells incubated for approximately 3 days to allow accumulation of the secreted protein in the medium. Harvested media was centrifuged and filtered and H_6_-tagged proteins recovered by nickel chromatography. After washing of columns, proteins were eluted with imidazole and Zeba columns used for buffer exchange into Tris-buffered saline (TBS; 25mM Tris, 150mM NaCl) containing 10% glycerol (v/v). Protein purity was assessed by SDS polyacrylamide gel electrophoresis and Coomassie staining. Protein concentrations were determined by absorbance at 280nm using the protein extinction coefficients according to Edelhoch [26] with the revised extinction coefficients for W and Y of Pace et al [27], and proteins were stored at 4°C before use.

### Biolayer interferometry

Binding analysis was performed by biolayer interferometry using an Octet RED instrument. Assay buffer comprised of tris-buffered saline with 0.05% (v/v) Tween-20 and 1mg/ml BSA. AHC biosensors were hydrated and then coated with ACE2-Fc by dipping sensors into assay buffer containing 5μg/ml ACE2 for 150 sec. After washing, association was measured by immersion of coated sensors in assay buffer containing soluble RBD monomers. For binding to human ACE2 RBD concentrations were 12, 30, 60 and 120nM and additionally 6nM in some cases: for rat ACE2 RBD concentrations were 48, 90, 150, 300 and 600nM and where no binding was detected an additional 1200nM was also tested. Data was analysed using the Octet Data Analysis Software with a 1:1 binding model and global fitting. In all cases global fitted curves had an R^2^ of >0.95 and χ^2^ of <0.05. All data are from at least three independent experiments for each RBD.

### Flow cytometry

Vero-E6 cells were cultured in DMEM with 10% (v/v) FBS. For cellular binding analysis cells were collected by centrifugation, washed and incubated for 15 min at 37°C with varying concentrations (as detailed in Results) of WH-RBD or S477N/Q498H-RBD in PBS with 10% (v/v) FBS. After washing bound RBD was detected by incubating cells for 10 min at room temperature with anti-HA conjugated to PE. Cells were then washed and mean fluorescence of stained cell populations determined on a FACS CantoII flow cytometer (Becton Dickinson).

### Complex preparation

The complex of ACE2 and mutant RBD used for cryo-EM was formed at room temperature for 30 min, concentrated using an Amicon Ultracel 10K Centrifugal Filter, cleared through a Millipore 0.22 micron Durapore centrifugal filter and applied to a Superdex-200 (10/300 GL) column (GE Healthcare) which was pre-equilibrated and then run in gel filtration buffer (TBS). Fractions were collected every 250 μl, monitored by *A*_230_ and analysed by SDS-PAGE (NuPAGE 4–12% Bis-Tris). Fractions containing the complex were selected and protein concentration determined using the extinction coefficient for the 1:1 complex at *A*_280_. The fraction containing the highest concentration of complex was then used for subsequent analysis by Cryo-EM.

### Cryo-electron microscopy

Grids were prepared on either unsupported holey grids or holey grids overlaid with graphene oxide. Samples derived from size exclusion chromatography were used without further concentration at 0.2mg/ml on holey grids or diluted to 0.05 mg/ml for graphene oxide grids. For the former, grids were glow-discharged for 60 sec at 35 mA on a Quorum GloQube. Graphene oxide grids were prepared as described before [24] and were glow-discharged at 40 mA for 180 sec prior to graphene oxide application. In each case 3μl of the complex was applied to grids (Quantifoil R1.2/1.3 300 mesh Au) and plunge frozen using a Thermo Fisher Scientific Vitrobot MKIV. A wait time of 30 sec was applied for graphene oxide grids to allow particles to adhere to the support film.

Data were collected on a Thermo Fisher Scientific Titan Krios G3 operating at 300 KeV and equipped with a Gatan BioQuantum energy filter with a slit width of 20 eV and a K3 direct electron detector. Movies were recorded at a nominal magnification of 105Kx in Counting Bin1 mode using aberration free image shift (AFIS). A total dose of 50e-/Å^2^ fractionated over 50 frames and a defocus range of -0.7μm to -2.7μm in 0.3 intervals were used. We proceeded to image the complex by Cryo-EM but initial attempts using holey grids resulted in strong preferential orientation and poor-quality maps (S2a Fig). When the complex was imaged on grids overlaid with graphene oxide we also observed a strong orientational bias in a different direction (S2b Fig). Combining the two datasets however resulted in a good quality 3.2Å map (Fig 3a).

### Image processing and model building

All data processing was performed using Relion 3 [28]. Briefly, movies were corrected for motion using MotionCor 2.1.4 [29] and the contrast transfer function parameters were estimated using GCTF 1.18 [30]. Particles were picked automatically using Topaz [31] initially with the supplied model but later on using a trained model based on the data. Initial processing of the unsupported dataset indicated severe preferential orientation (S2 Fig) and despite efforts to computationally balance the dataset a good 3D map could not be obtained. A second dataset on graphene oxide also indicated strong preferential orientation in a different direction so the two datasets, collected under identical optics conditions, were combined and processed together. After several rounds of cleanup using 2D classification, the data were subjected to 3D classification and the best subset was chosen for further refinement. The data was further improved by refining CTF parameters and aberrations and particle polishing. A final map was obtained at a global resolution of 3.2Å using the Gold Standard FSC 0.143 criterion.

Initial rigid-body docking of the crystal structure (PDB ID 6M0J) was performed using UCSF Chimera 1.15 [32] and further model building was performed in Coot 0.9.6 [33]. After manual rebuilding real-space refinement of the coordinates was performed using Phenix 1.19.2 [34]. All figures were created sing Chimera X 1.3rc [35].

The Cryo-EM maps and coordinates have been deposited to the EMDB (EMD-14666) and PDB (PDB ID 7ZDQ) respectively.

## Supporting information

S1 Fig Example of multiple conformations of Q498 suggested in crystal structures. The 2Fo-Fc electron density map (in blue, contoured at 1.5σ) and Fo-Fc difference map (contoured at -3σ (red) and green (+3σ)) are shown for the WT crystal structure of ACE2-RBD (PDB ID 6M0J [13]). The positive (green) density adjacent to the side chain of Q498 suggests this can exist in different conformations, thus weakening the interaction with neighbouring residues. Examination of the region of Q498 in PDB entries 7WQB, 7RPV, 7EFR, 7EFP, 7NXC, 7L0N, 7DMU and 6VW1 all show difference electron density adjacent to the side chain. PDB entries 7EKE, 7EKY, 7EKH, 7EKF and 6LZG all have the residue modelled in dual conformations. Only 7LO4 does not show disorder in Q498.
(DOCX)

S2 Fig Data processing outline. Datasets were collected on both holey and graphene oxide support films. Each dataset present a different angular bias so the two datasets were combined. Following clean-up by 2D classification the particles were subjected to 3D classification and the single best 3D class chosen for further refinement and polishing to produce the final map.
(DOCX)

S3 Fig Angular distribution and resolution. The angular distribution of the final map is shown in (a) with an overall uniform angular distribution despite some persistent angular bias. The local resolution map is shown in (b) and the FSC of the two half maps and map vs model are shown in (c).
(DOCX)

S1 Table Cryo-EM data collection, refinement and validation statistics. (DOC)
