# Supplementary material for: In vitro evolution predicts emerging SARS-CoV-2 mutations with high affinity for ACE2 and cross-species binding
Source: PLoS Pathog. 2022 Jul 18;18(7):e1010733. doi: 10.1371/journal.ppat.1010733 (PMC9333441; doi:10.1371/journal.ppat.1010733)
Supplement: S1 Fig — The 2Fo-Fc electron density map (in blue, contoured at 1.5σ) and Fo-Fc difference map (contoured at -3σ (red) and green (+3σ)) are shown for the WT crystal structure of ACE2-RBD (PDB ID 6M0J [13]). The positive (green) density adjacent to the side chain of Q498 suggests this can exist in different conformations, thus weakening the interaction with neighbouring residues. Examination of the region of Q498 in PDB entries 7WQB, 7RPV, 7EFR, 7EFP, 7NXC, 7L0N, 7DMU and 6VW1 all show difference electron density adjacent to the side chain. PDB entries 7EKE, 7EKY, 7EKH, 7EKF and 6LZG all have the residue modelled in dual conformations. Only 7LO4 does not show disorder in Q498. (DOCX) [file ppat.1010733.s001.docx]

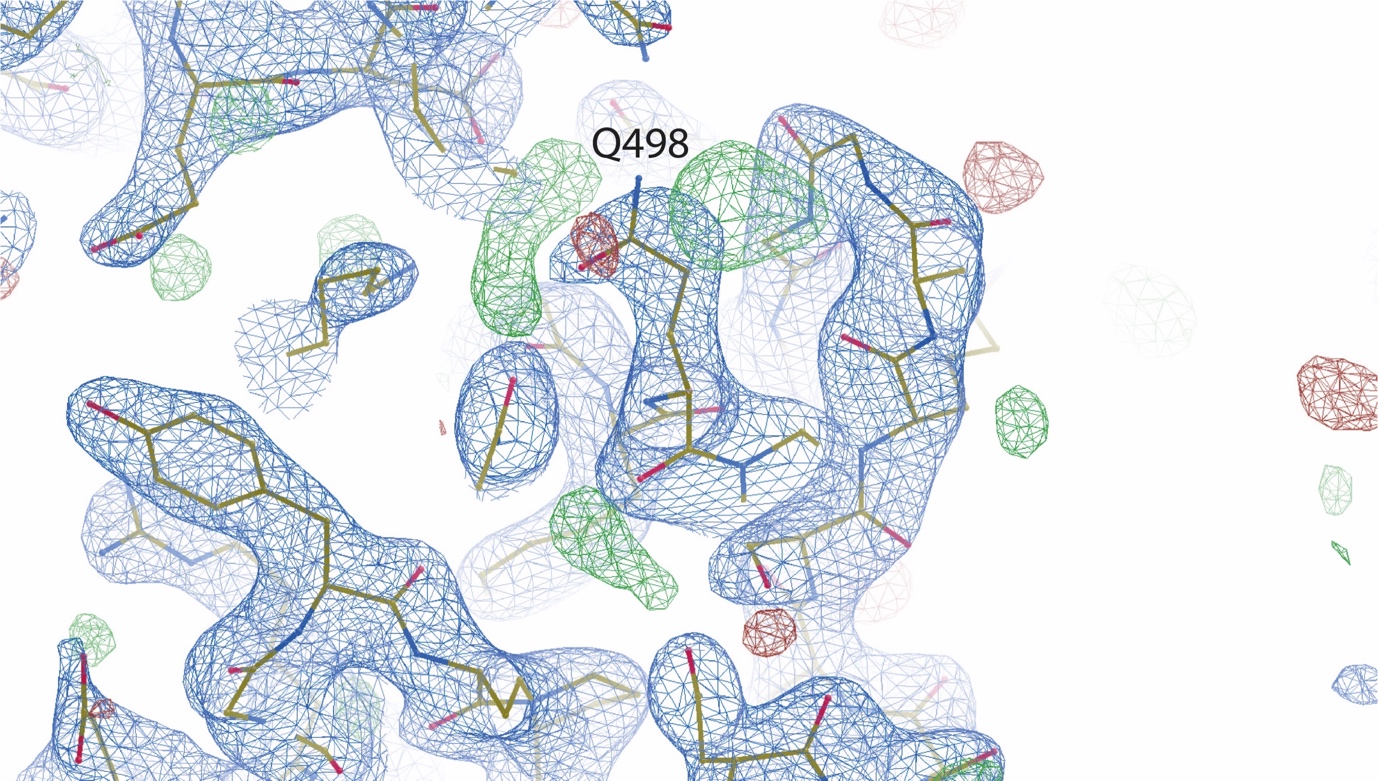


**S1 Fig.** **Example of** **multiple conformations of Q498 suggested in crystal structures.** The 2Fo-Fc electron density map (in blue, contoured at 1.5σ) and Fo-Fc difference map (contoured at -3σ  (red) and green (+3σ)) are shown for the WT crystal structure of ACE2-RBD (PDB ID 6M0J [13]).  The positive (green) density adjacent to the side chain of Q498 suggests this can exist in different conformations, thus weakening the interaction with neighbouring residues. Examination of the region of Q498 in PDB entries 7WQB, 7RPV, 7EFR, 7EFP, 7NXC, 7L0N, 7DMU and 6VW1 all show difference electron density adjacent to the side chain. PDB entries 7EKE, 7EKY, 7EKH, 7EKF and 6LZG all have the residue modelled in dual conformations. Only 7LO4 does not show disorder in Q498.
