# Supplementary material for: In vitro evolution predicts emerging SARS-CoV-2 mutations with high affinity for ACE2 and cross-species binding
Source: PLoS Pathog. 2022 Jul 18;18(7):e1010733. doi: 10.1371/journal.ppat.1010733 (PMC9333441; doi:10.1371/journal.ppat.1010733)
Supplement: S2 Fig — Datasets were collected on both holey and graphene oxide support films. Each dataset present a different angular bias so the two datasets were combined. Following clean-up by 2D classification the particles were subjected to 3D classification and the single best 3D class chosen for further refinement and polishing to produce the final map. (DOCX) [file ppat.1010733.s002.docx]

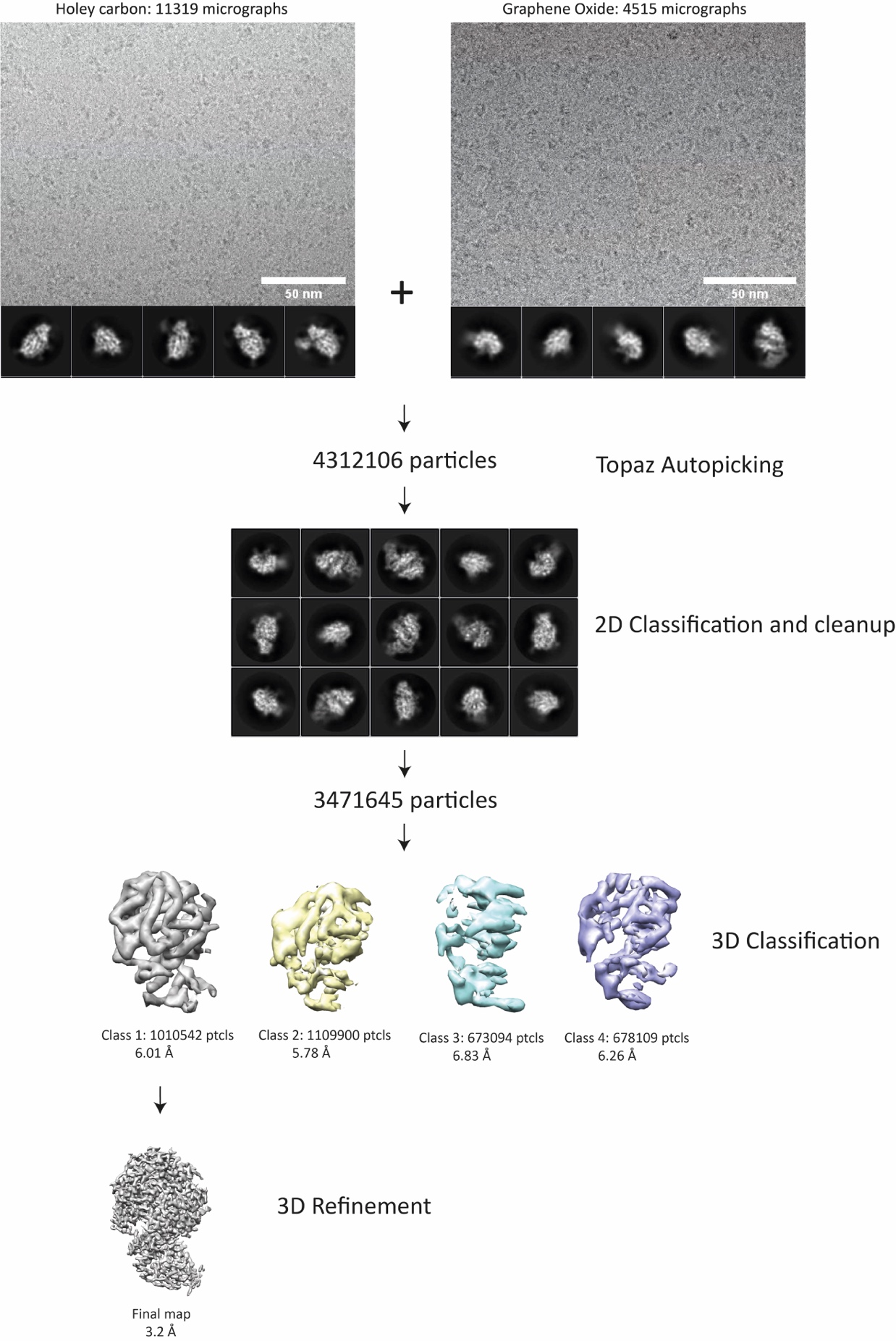


**S2 Fig. Data processing outline:** Datasets were collected on both holey and graphene oxide support films. Each dataset presents a different angular bias so the two datasets were combined. Following clean-up by 2D classification the particles were subjected to 3D classification and the single best 3D class chosen for further refinement and polishing to produce the final map.
