# Supplementary material for: In vitro evolution predicts emerging SARS-CoV-2 mutations with high affinity for ACE2 and cross-species binding
Source: PLoS Pathog. 2022 Jul 18;18(7):e1010733. doi: 10.1371/journal.ppat.1010733 (PMC9333441; doi:10.1371/journal.ppat.1010733)
Supplement: S3 Fig — The angular distribution of the final map is shown in (a) with an overall uniform angular distribution despite some persistent angular bias. The local resolution map is shown in (b) and the FSC of the two half maps and map vs model are shown in (c). (DOCX) [file ppat.1010733.s003.docx]

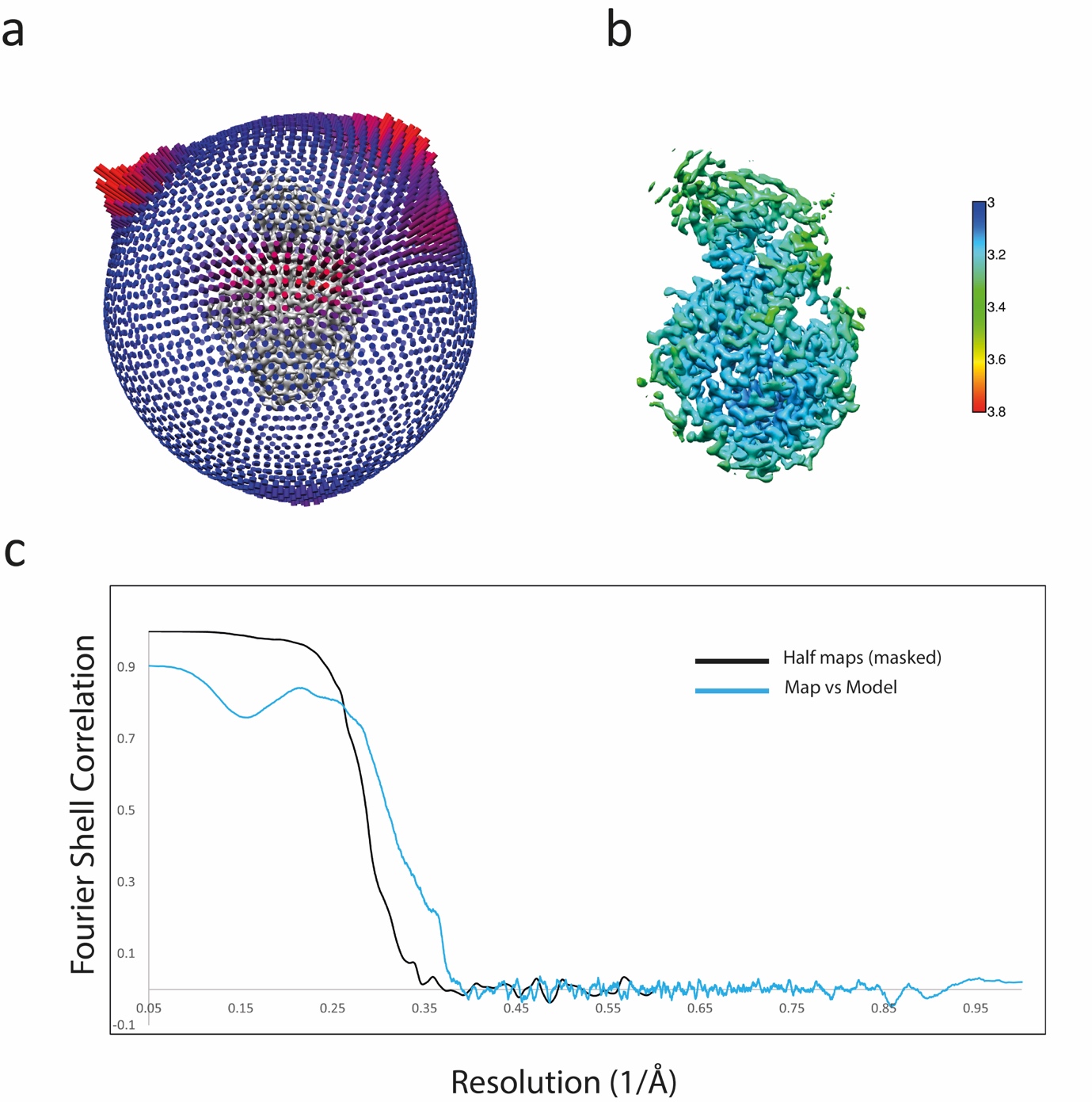


**S3 Fig. Angular distribution and resolution:** The angular distribution of the final map is shown in (a) with an overall uniform angular distribution despite some persistent angular bias. The local resolution map is shown in (b) and the FSC of the two half maps and map vs model are shown in (c).
