# Supplementary material for: In vitro evolution predicts emerging SARS-CoV-2 mutations with high affinity for ACE2 and cross-species binding
Source: PLoS Pathog. 2022 Jul 18;18(7):e1010733. doi: 10.1371/journal.ppat.1010733 (PMC9333441; doi:10.1371/journal.ppat.1010733)
Supplement: S1 Table — (DOC) [file ppat.1010733.s004.doc]

**S1 Table. Cryo-EM data collection, refinement and validation statistics**

|  | ACE2-RBD  (EMDB-14666)  (PDB 7ZDQ) |  |
| --- | --- | --- |
| **Data collection and processing** |  |  |
| Magnification | 105,000 |  |
| Voltage (kV) | 300 |  |
| Electron exposure (e–/Å^2^) | 50 |  |
| Defocus range (μm) | -0.7 to -2.7 |  |
| Pixel size (Å) | 0.835 |  |
| Symmetry imposed | C1 |  |
| Initial particle images (no.) | 4,312,106 |  |
| Final particle images (no.) | 1,010,542 |  |
| Map resolution (Å)  FSC threshold | 3.2  0.143 |  |
| Map resolution range (Å) | 4.0-3.0 |  |
|  |  |  |
| **Refinement** |  |  |
| Initial model used (PDB code) | 6M0J |  |
| Model resolution (Å)  FSC threshold | 3.2  0.143 |  |
| Map sharpening *B* factor (Å^2^) | -162 |  |
| Model composition  Non-hydrogen atoms  Protein residues  Ligands | 6264  763  3 |  |
| *B* factors (Å^2^)  Protein  Ligand | 28.46  46.36 |  |
| R.m.s. deviations  Bond lengths (Å)  Bond angles (°) | 0.004  0.678 |  |
| Validation  MolProbity score  Clashscore  Poor rotamers (%) | 1.41  6.38  0.15 |  |
| Ramachandran plot  Favored (%)  Allowed (%)  Disallowed (%) | 97.75  2.25  0.00 |  |
